# Effects of veverimer on serum bicarbonate and physical function in women with chronic kidney disease and metabolic acidosis: a subgroup analysis from a randomised, controlled trial

**DOI:** 10.1186/s12882-022-02690-1

**Published:** 2022-02-25

**Authors:** Vandana S. Mathur, Donald E. Wesson, Navdeep Tangri, Elizabeth Li, David A. Bushinsky

**Affiliations:** 1 MathurConsulting LLC, 25 Upenuf Road, Suite 100, Woodside, CA 94062-2633 USA; 2 Texas A&M Health Sciences Center College of Medicine, Dallas, TX USA; 3 Donald E Wesson Consulting, LLC, Dallas, TX USA; 4 University of Manitoba, Winnipeg, MB Canada; 5 Pharmastat LLC, Fremont, CA USA; 6 University of Rochester School of Medicine and Dentistry, Rochester, NY USA

**Keywords:** Chronic kidney disease, Metabolic acidosis, Disparity, Sex, Women, Serum bicarbonate, Veverimer, Physical function

## Abstract

**Background:**

Globally, the prevalence of chronic kidney disease (CKD) is higher in women than in men; however, women have been historically under-represented in nephrology clinical trials. Metabolic acidosis increases risk of progressive loss of kidney function, causes bone demineralization and muscle protein catabolism, and may be more consequential in women given their lower bone and muscle mass. Veverimer, an investigational, non-absorbed polymer that binds and removes gastrointestinal hydrochloric acid, is being developed as treatment for metabolic acidosis.

**Methods:**

This was a Phase 3, multicenter, randomised, blinded, placebo-controlled trial in 196 patients with CKD (eGFR: 20–40 mL/min/1.73 m^2^) and metabolic acidosis who were treated for up to 1 year with veverimer or placebo. We present the findings from a pre-specified subgroup analysis evaluating the effects of veverimer on metabolic acidosis and physical function among women (*N* = 77) enrolled in this trial.

**Results:**

At week 52, women treated with veverimer had a greater increase in mean (± standard error) serum bicarbonate than the placebo group (5.4 [0.5] vs. 2.2 [0.6] mmol/L; *P* < 0.0001). Physical Function reported by patients on the Kidney Disease and Quality of Life – Physical Function Domain, a measure that includes items related to walking, stair climbing, carrying groceries and other activities improved significantly in women randomized to veverimer vs placebo (+ 13.2 vs. -5.2, respectively, *P* < 0.0031). Objectively measured performance time on the repeated chair stand test also improved significantly in the veverimer group vs. placebo (*P* = 0.0002).

**Conclusions:**

Veverimer was effective in treating metabolic acidosis in women with CKD, and significantly improved how they felt and functioned.

**Trial registration:**

ClinicalTrials.gov Identifier: NCT03390842. Registered on January 4, 2018.

**Supplementary Information:**

The online version contains supplementary material available at 10.1186/s12882-022-02690-1.

## Introduction

Globally, the prevalence of chronic kidney disease (CKD) is higher in women than in men with a ratio of approximately 4:3 [1]. The historic under-representation of women in nephrology clinical trials may not allow for adequate generalization of findings to women [2]. Thus, evaluation of findings from clinical trials within the subgroup of women is important.

Metabolic acidosis develops in CKD because of acid retention from impaired kidney acid excretion. The inability to excrete acid is primarily due to reduced ammoniagenesis and impaired ammonium excretion [3]. Metabolic acidosis increases the risk of progression of CKD and leads to catabolism of muscle protein and loss of muscle mass [4]. Acidemia directly stimulates glutamine extraction from blood by several-fold [5] and increases proximal tubule glutamine catabolism – a process that generates new bicarbonate. The bicarbonate enters the systemic circulation, whereas ammonia is excreted into the urine after titration to ammonium [6]. The high demand for glutamine to support maximal ammoniagenesis by the nephrons is met, in part, by metabolic acidosis-induced catabolism of skeletal muscle protein [5]. Skeletal muscle protein catabolism allows for maximal acid excretion.

In women, the combined effects of older age, gender, and post-menopausal status may synergize with the catabolic effects of metabolic acidosis. Moreover, the muscular effects of metabolic acidosis may be more consequential in causing functional decline in women given their lower baseline muscle mass [7, 8]. Alterations in sex hormone levels related to age and/or disease states are major contributors to muscle wasting. As such, men and women may respond differently to catabolic conditions, including metabolic acidosis [7]. With aging, reductions in muscle mass, muscle strength, and physical function and increase in fat mass are more prominent in men; however, the sarcopenia prevalence is higher in women [7]. Further, the functional implications of sarcopenia are also greater in women. For all activities (functional activities [i.e., lifting, climbing stairs, walking, sustained standing, bending, reaching, and grasping], activities of daily living, and instrumental activities of daily living) and across all age groups, the prevalence of disability was greater for women than for men based on a national interview study [9]. The effect of treating metabolic acidosis on physical function in women with CKD has not been previously well described.

Veverimer is an orally administered, non-absorbed, polymer drug that increases serum bicarbonate by selectively binding protons and chloride in the gastrointestinal tract and removing this bound hydrochloric acid (HCl) via fecal excretion [10]. In a prior 2-week study of men and women with CKD and metabolic acidosis conducted in an inpatient research unit, veverimer significantly increased serum bicarbonate within 24 h following the first dose. After 2 weeks of treatment the serum bicarbonate had increased by 3–4 mmol/L [11]. However, the differential effects of veverimer on serum bicarbonate levels and muscle function in a larger multicenter randomized controlled trial by sex are unknown.

Here we report further details of a pre-specified subgroup analysis of women from a previously published multicenter, randomized, controlled trial [12].

## Methods

Methods for this study have been previously reported [12] and are briefly summarized below.

### Study design

This was a multicenter, randomised, blinded, placebo-controlled 40-week extension study of our 12-week parent study [13] conducted at 29 sites in 7 countries [12]. The study was registered on ClinicalTrials.gov (NCT03390842) on January 4, 2018. The study protocol was approved by each site’s institutional review board or ethic committee and appropriate regulatory authorities. Each patient gave his or her written informed consent prior to participation in the trial. Patients who continued from the parent study into the extension study did so with no gap in their study treatment and they continued the same blinded treatment they had received in the parent study. Following enrollment, scheduled visits were conducted at weeks 14, 16, 20, 24, 28, 34, 40, 46, and 52 (Supplemental Fig. 1). The study was stopped once the last patient had completed the final study assessment.

### Patients

Patients with CKD (estimated glomerular filtration rate [eGFR] 20–40 mL/min/1.73 m^2^) and metabolic acidosis (serum bicarbonate 12–20 mmol/L) were enrolled into the parent study and randomised 4:3 to veverimer or placebo by an interactive web-based response system. Eligibility was based on three qualifying bicarbonate values and two qualifying screening eGFR values not different by more than 20% and in the range of 20–40 mL/min per 1.73 m^2^. Hemoglobin A1c at screening was required to be ≤9.0%. Eligibility for the extension study required completion of the 12-week parent study. Patients were excluded from participation if they had a serum bicarbonate concentration low enough to need emergency intervention or had an assessment for an acute acidotic process; required dialysis for acute kidney injury or worsening CKD during the parent study; planned kidney replacement therapy within 6 months; had clinically significant gastroparesis, bariatric surgery, bowel obstruction, swallowing disorders, severe gastrointestinal disorders, inflammatory bowel disease, major gastrointestinal surgery, or active gastric or duodenal ulcers, or both. The full eligibility criteria have been previously reported [12].

### Procedures

The starting study drug dose in the parent study was 6 g of veverimer once daily (two packets per day) or placebo once daily (2 packets per day). Both were administered orally as a suspension in 60 mL of water. The study drug dose was algorithmically titrated by the interactive response technology system in the range of 0–9 g/day (or equivalent number of placebo packets) to a target serum bicarbonate concentration of 22–29 mmol/L based on bicarbonate measurement at each visit. Background use of oral alkali supplements was permitted at a stable dose in the parent study and continued into the extension study. To avoid the long-term sodium or potassium load with oral alkali treatment in the extension study, the alkali dose was discontinued once the serum bicarbonate increased to ≥22 mmol/L. There were no protocol-specified dietary restrictions. Dietary counselling was provided to patients in accordance with dietary recommendations for patients with CKD (e.g., Kidney Disease Improving Global Outcome [KDIGO] 2013 [14]). Bicarbonate measurements were made using a calibrated iSTAT Handheld Blood Analyzer (Abbott Point of Care, Princeton, NJ, USA); the total CO_2_ was calculated. “Serum bicarbonate” in this study thus refers to the total CO_2._ All other clinical laboratory measurements were done by a central laboratory. Management of blood pressure and glycemic control was at the discretion of the investigator.

The Kidney Disease and Quality of Life Short Form-36, question 3 (Physical Function Domain; KDQoL-PFD) and standardized repeated chair stand test were administered at baseline and weeks 12, 40, and 52. The KDQoL-PFD (Supplemental Table 1) was forward and backwards translated, linguistically validated (including clinician’s review), and culturally adapted. The paper questionnaires, consisting of 10 questions, were completed by patients by themselves, while at the study site. Patients responded to the question: “The following items are about activities you might do during a typical day. Does your health now limit you in the activities? If so, how much?”. Answer choices were “yes, limited a lot”, “yes, limited a little”, and “no, not limited at all”.

The five-times repeated chair stand test, a component of the Short Physical Performance Battery, was administered by study site personnel using a verbatim written script (in the patient’s spoken language) to instruct patients during the test. The time for a patient to complete five repeated sit-stands with arms folded across the chest from an armless chair was measured with a stopwatch.

The primary endpoint for the extension study was the long-term safety based on the incidence of adverse events (AEs), serious AEs (SAEs), and AEs leading to withdrawal. Secondary endpoints (analyzed in pre-specified rank order) compared veverimer to placebo at Week 52: achieving a ≥ 4 mmol/L increase from baseline in serum bicarbonate or a serum bicarbonate in the normal range (22–29 mmol/L); the change from baseline in serum bicarbonate to Week 52; the change from baseline in total KDQoL-PFD score; and the change from baseline in the time to complete the repeated chair stand test. Baseline serum bicarbonate was determined in the parent study as the mean of the serum bicarbonate values from screening 1, screening 2, and day 1 (pre-dose) visits. Baseline values of total KDQoL-PFD score and repeated chair stand test were the measurements taken at the day 1 (pre-dose) visit in the parent study.

Adverse events were identified by several methods. Patients were questioned at every study visit about any adverse effects they had experienced. Additionally, investigators were required to report any adverse events revealed from physical examination, laboratory tests, ECG findings, and other assessments.

The study patients were required to return all used and unused packets of the study drug at each visit. The compliance was calculated based on the returned empty packets and expected usage.

Additional details related to study procedures have been previously reported [12].

### Statistical methods

The safety analysis set was defined as all patients who received any amount of study drug (veverimer or placebo) in the extension study and was used for assessments of safety. A modified intention-to-treat analysis set, defined as all randomly assigned patients who had both baseline and at least one post-baseline serum bicarbonate value in the parent study and at least one serum bicarbonate value after the week 12 visit in the extension study, was used for evaluation of efficacy (secondary endpoints), based on planned treatment assignment. To control family-wise error rate, hypothesis testing for the 4 durability-of-effect (secondary) endpoints was prespecified to be done sequentially, with subsequent tests only done when all previous tests were statistically significant at the two-sided 0.05 level: responder analysis at week 52 using the Fisher’s exact test; change from baseline to week 52 in serum bicarbonate using a mixed model for repeated measurements; change from baseline to week 52 in the total KDQoL-PFD score using a rank-based ANCOVA model; and change from baseline to week 52 in the duration of the repeated chair stand test using a rank-based ANCOVA model. Additional details related to statistical methods have been previously reported [12].

To evaluate the overall effect of sex on the efficacy of veverimer, we performed exploratory analyses for each of the 4 efficacy endpoint analyses. These analyses were performed using a logistic regression model for the composite (dichotomous) endpoint, a mixed effect model for the change from baseline in serum bicarbonate endpoint, and rank-based ANCOVA models for the KDQoL and repeated chair stand test endpoints. These exploratory analysis models included two additional fixed effects: sex and treatment by sex interaction.

## Results

Results for the overall study population have been previously reported [12].

### Baseline characteristics

**Fig. 1:**
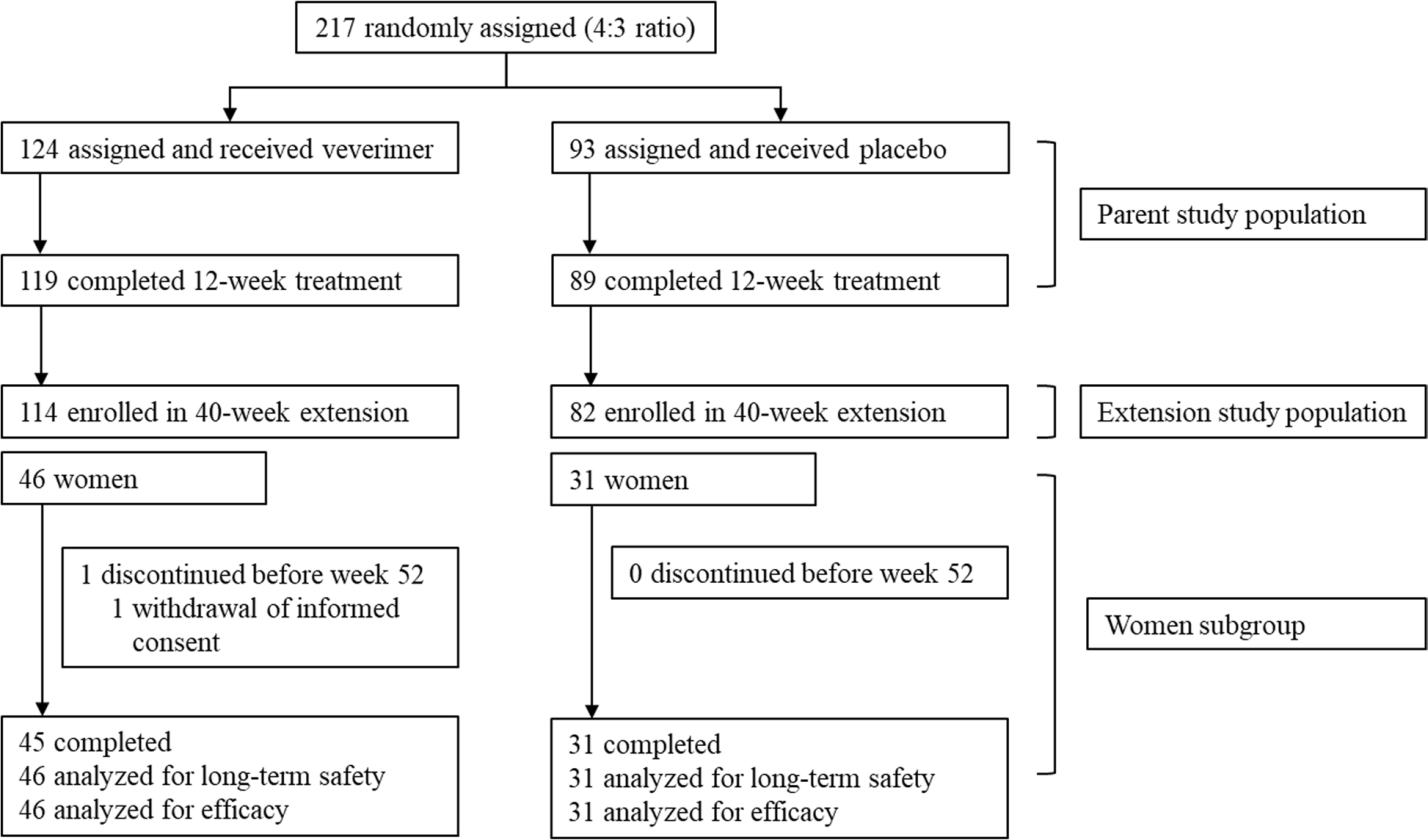
Participant Flow for Women Subgroup. In the parent study, 5 patients discontinued treatment in the veverimer group (1 dialysis, 1 adverse event, 3 withdrawal or discontinuation for other reasons) and 4 patients discontinued treatment in the placebo group (2 died, 1 adverse event, and 1 withdrawal) before week 12. Five patients in the veverimer group and 6 patients in the placebo group declined to participate in the extension study, and one patient in the placebo group was ineligible for participation in the extension study. In the overall extension study, 3 patients discontinued treatment in the veverimer group (1 withdrawal and 2 lost to follow-up) and 8 patients discontinued treatment in the placebo group (2 died, 1 dialysis, 3 withdrawals, 1 lost to follow-up, and 1 other)

Of the 217 randomised patients in the 12-week parent study, 196 patients were enrolled in the 40-week extension study, 77 of whom were women (46 in the veverimer group and 31 in the placebo group) (Fig. 1). In the veverimer and placebo groups, respectively, 97.3% (111/114) and 90.0% (74/82) of patients completed the study. The mean (standard deviation) daily dose in the veverimer group was 7.9 (1.8) g/day. Patients were defined as having dosing compliance if they took > 80% of the prescribed doses. Compliance with dosing was achieved in 100% of patients in the veverimer group and 99% of patients in the placebo groups.

Baseline characteristics within the subgroup of women and the overall study population, including demographics, serum bicarbonate, eGFR, and the urine albumin to creatinine ratio were generally balanced across treatment groups; however, among women patients, patient-reported physical function (KDQoL-PFD) was numerically lower in the veverimer group (48.4 points) compared with the placebo group (58.2 points) (Table 1). Among all women, the mean age was 65.4 years, the mean baseline eGFR was 28.4 mL/min/1.73 m^2^, the mean baseline serum bicarbonate was 17.3 mmol/L, and 9.1% were on background oral alkali.

**Table 1:** Baseline demographic and clinical characteristics

Parameter	Overall Population		Subgroup of Women	
	Veverimer (***N*** = 114)	Placebo (***N*** = 82)	Veverimer (***N*** = 46)	Placebo (***N*** = 31)
Age (y), mean (SD)	62.9 (12.1)	61.7 (11.9)	65.7 (10.5)	65.0 (7.2)
Age ≥ 65 years	58 (51)	38 (62)	25 (54)	17 (55)
Sex (female), n (%)	46 (40)	31 (38)	46 (100)	31 (100)
Race (White), n (%)	113 (99)	79 (96)	45 (98)	30 (97)
Body mass index (kg/m^2^)	28.7 (4.0)	27.9 (3.9)	28.5 (3.5)	27.8 (3.2)
SBP (mmHg), mean (SD)	135.9 (8.9)	136.5 (9.0)	133.4 (7.1)	134.9 (7.4)
Selected medical history, n (%)				
Hypertension	110 (96)	79 (96)	44 (96)	29 (94)
Congestive heart failure	34 (30)	28 (34)	10 (22)	13 (42)
Left ventricular hypertrophy	56 (49)	35 (43)	19 (41)	14 (45)
Diabetes	70 (61)	57 (70)	27 (59)	22 (71)
Myocardial infarction	17 (15)	10 (12)	5 (11)	2 (7)
Percutaneous coronary intervention or coronary bypass graft	19 (17)	14 (17)	5 (11)	3 (10)
Peripheral vascular disease	5 (4)	6 (7)	1 (2)	2 (7)
Stroke	8 (7)	8 (10)	3 (7)	3 (10)
Laboratory values, mean (SD)				
Serum bicarbonate (mmol/L)	17.2 (1.4)	17.1 (1.5)	17.2 (1.5)	17.3 (1.3)
eGFR (mL/min/1.73 m^2^)	29.4 (6.4)	27.9 (5.4)	28.5 (5.6)	28.2 (4.9)
Serum potassium (mmol/L)	4.9 (0.6)	4.9 (0.6)	4.9 (0.7)	4.7 (0.4)
Hemoglobin A1c (%)	6.1 (0.9)	6.2 (1.1)	6.0 (1.2)	6.2 (1.2)
Serum albumin (g/dL)	4.1 (0.4)	4.0 (0.3)	4.1 (0.4)	4.1 (0.3)
ACR (mg/g), geometric mean (95% CI)	209 (147, 297)	305 (207, 449)	127 (71, 229)	204 (111, 376)
ACR > 300 mg/g, n (%)	50 (47)	49 (65)	16 (36)	11 (36)
Hemoglobin (g/dL), mean (SD)	12.6 (1.8)	12.6 (1.7)	12.1 (1.4)	12.2 (1.7)
Concomitant medications, n (%)				
ACE inhibitor or ARB	75 (67)	66 (82)	29 (63)	23 (74)
Sodium bicarbonate	11 (10)	5 (6)	3 (10)	4 (9)
Physical functioning, mean (SD)				
KDQOL-PFD total score	52.6 (22.4)	55.7 (26.2)	48.4 (21.9	58.2 (22.3)
Repeated chair stand (s)	21.7 (16.9)	21.0 (17.1)	21.7 (18.6)	21.5 (17.2)

*ACR* Urine albumin to creatinine ratio, *eGFR* Estimated glomerular filtration rate, *KDQOL-PFD* Kidney Disease Quality of Life physical function domain, *SBP* Systolic blood pressure

In the parent study, 5 patients discontinued treatment in the veverimer group (1 dialysis, 1 adverse event, 3 withdrawal or discontinuation for other reasons) and 4 patients discontinued treatment in the placebo group (2 died, 1 adverse event, and 1 withdrawal) before week 12. Five patients in the veverimer group and 6 patients in the placebo group declined to participate in the extension study, and one patient in the placebo group was ineligible for participation in the extension study. In the overall extension study, 3 patients discontinued treatment in the veverimer group (1 withdrawal and 2 lost to follow-up) and 8 patients discontinued treatment in the placebo group (2 died, 1 dialysis, 3 withdrawals, 1 lost to follow-up, and 1 other).

### Effect of veverimer on serum bicarbonate levels

**Fig. 2:**
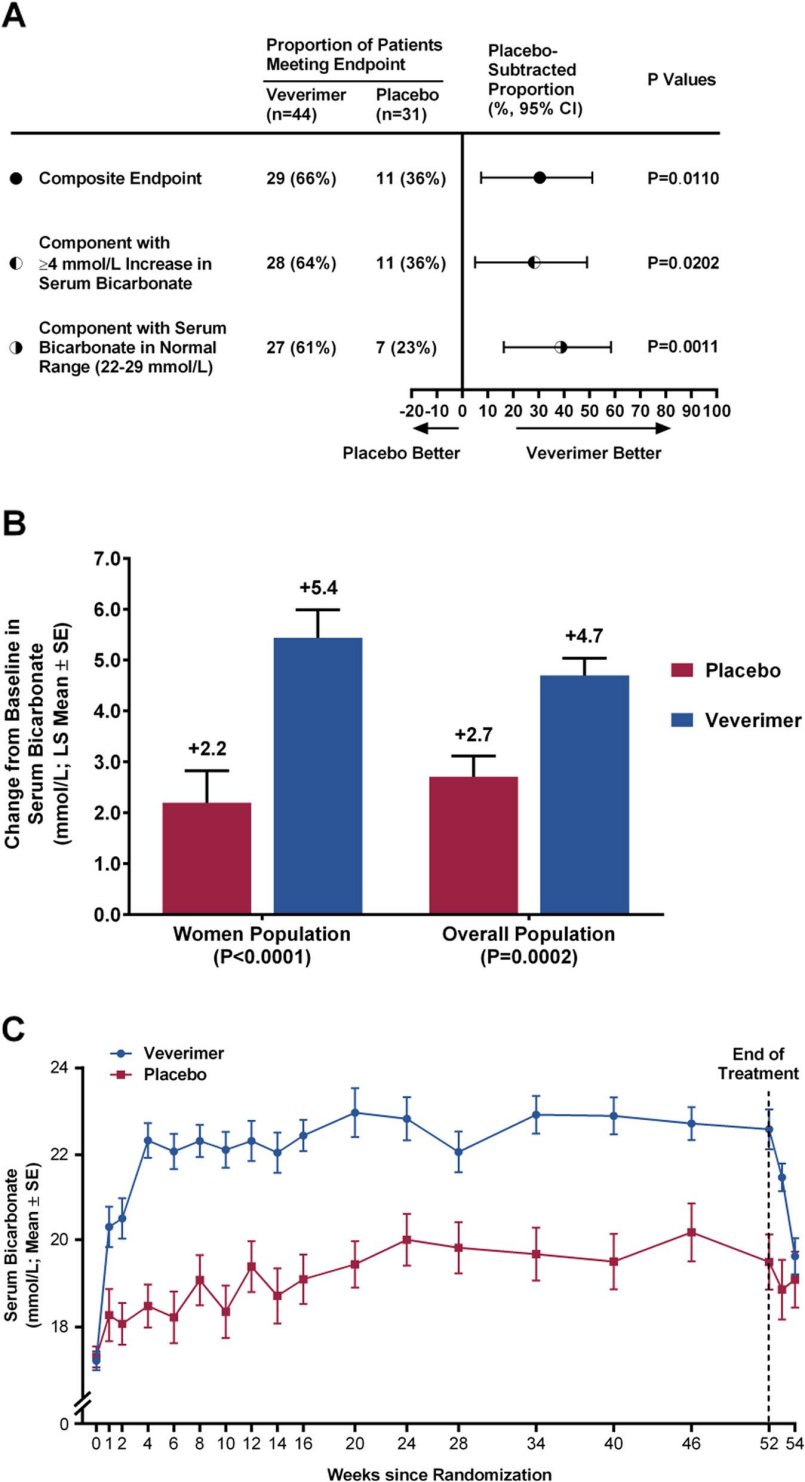
Veverimer Effects on Serum Bicarbonate. **A** The top line shows the composite endpoint at treatment week 52. The two lower lines depict each component of the primary endpoint (percentage of patients who had a ≥ 4 mmol/L increase or normalization of serum bicarbonate at week 52). *P*-values are for the difference in proportions between the veverimer and placebo groups. **B** Change in serum bicarbonate from baseline to week 52. **C** Serum bicarbonate levels over time. The baseline serum bicarbonate was 17.2 (0.2) mmol/L and 17.3 (0.2) mmol/L in the veverimer and placebo groups, respectively. LS, least squares; SE, standard error

A significantly greater percentage of women in the veverimer group at Week 52 met the composite endpoint (a ≥ 4 mmol/L increase or normalization of serum bicarbonate) compared with the placebo group (66% vs. 36%, *P* = 0.011) (Fig. 2A). Additionally, the increase from baseline in serum bicarbonate in patients in the veverimer group was significantly greater than in the placebo group (least squares mean increase of 5.4 [0.5] vs. 2.2 [0.6] mmol/L, *P* < 0.0001) (Fig. 2B). These findings were consistent with those from the overall study population (Fig. 2B). The significant effect of veverimer on serum bicarbonate was observed within 1 week of the first dose and was maintained through Week 52, the end of treatment (Fig. 2C). The effects of veverimer were similar in the subgroup of patients on proton pump inhibitors or H2 receptor blockers compared with the effects in the overall population [12].

For both the dichotomous and continuous variable bicarbonate-based endpoints, the treatment interaction for the variable “sex” was non-significant (*p* = 0.31 and *p* = 0.39, respectively), indicating that the effect of veverimer on serum bicarbonate did not vary significantly by sex (see also Supplement Fig. 2).

### Effects of veverimer on physical function

**Fig. 3:**
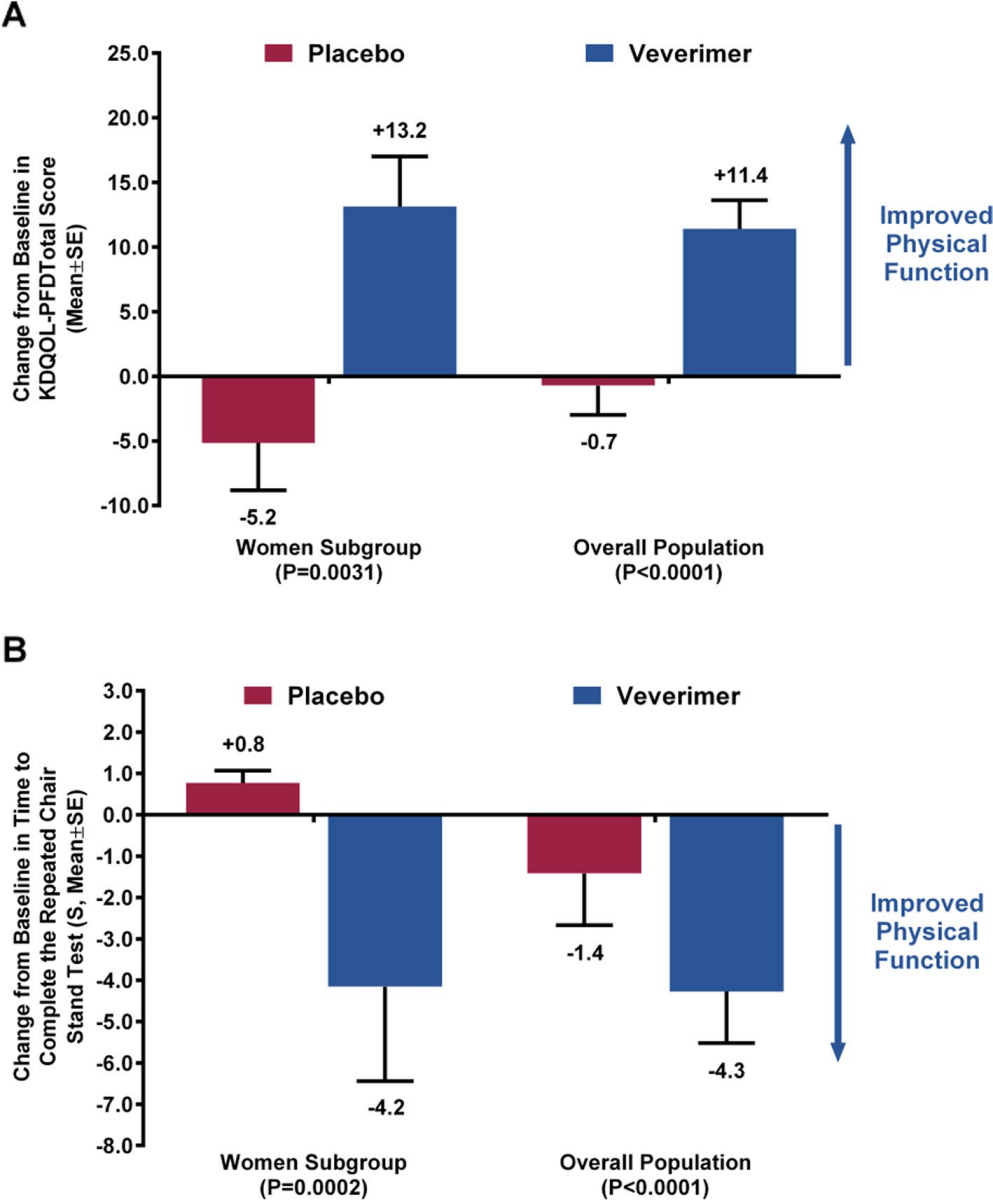
Veverimer Effects on Physical Function. **A** Change from baseline in KDQOL-PFD. **B** Change from baseline in time to complete the repeated chair stand test. KDQOL-PFD, Kidney Disease Quality of Life physical function domain; SE, standard error

In the subgroup of women, patient-reported limitations of physical function on the KDQoL-PFD, which measured daily activities such as walking, bending/stooping, and climbing stairs, improved significantly in the veverimer group vs. the placebo group (+ 13.2 vs. -5.2 points, respectively, *P* < 0.0031; Fig. 3A). The mean (SD) KDQoL-PFD score increased (indicating better functioning) in the veverimer group, from 48.4 (21.9) at baseline to 61.5 (21.5) at Week 52, but worsened in the placebo group (58.2 [22.3] to 53.1 [20.1]).

Additionally, physical performance, measured directly and objectively with the repeated chair stand test at Week 52 also improved to a significantly greater extent in the veverimer group compared with the placebo group (− 4.2 s vs. + 0.8 s, *P* < 0.0002; Fig. 3B). The mean (SD) chair stand time decreased (indicating better functioning) in the veverimer group, from 21.7 (18.6) seconds at baseline to 16.7 (10.7) seconds at Week 52, but was essentially unchanged in the placebo group (21.5 [17.2] to 22.2 [16.5] seconds). These findings were consistent with those observed in the overall study population (Fig. 3A and B).

The individual questions (items) of the KDQoL-PFD were also analyzed in the overall study population. There were significant improvements in climbing a flight of stairs, all items related to walking, and activities of bending/kneeling/stooping in the veverimer group compared to the placebo group [12]. Lower extremity strength is needed for these activities on which patients reported improvements as well as for time on the repeated chair stand test which also improved.

For both the KDQoL and repeated chair stand endpoints, the treatment interaction for the variable “sex” was non-significant (*p* = 0.32 and *p* = 0.37, respectively), indicating that the effect of veverimer on physical function did not vary significantly by sex.

### Safety of veverimer

In the overall study population, treatment for up to 1 year with veverimer was tolerated well and the safety profile was similar to that observed in the placebo group [12]. There were two deaths in the placebo group and none in the veverimer group. Fewer patients in the veverimer group than in the placebo group prematurely discontinued the study treatment (3% vs. 10%, respectively) and no patient in the veverimer group discontinued due to an adverse event (AE). Serious AEs were reported in 2% vs. 5% of patients in the veverimer and placebo groups, respectively. None of the SAEs were considered related to the study drug by the investigator. Headache was the only AE with a between-group difference of more than 5% and was more commonly reported in the placebo group [12]. All adverse events coded to the renal system in this study were related to worsening kidney function other than one event of proteinuria. These renal system adverse events were reported in 8 and 15% of patients in the veverimer group and the placebo groups, respectively. Only one patient (in the veverimer group) had a serum bicarbonate > 30 mmol/L during the study and this increase occurred in the context of over-diuresis. Veverimer showed no apparent off-target effects on other electrolytes, lipids, vital signs, or electrocardiogram intervals [12].

Among women, AEs were reported in 91 and 84% of patients in the veverimer and placebo groups, respectively (Table 2). Treatment-related AEs were reported in 42% of patients in the placebo group and in 26% of patients in the veverimer group (Table 2).

**Table 2:** Safety summary in the subgroup of women

	Veverimer (*n* = 46)	Placebo (*n* = 31)
Deaths	0	0
Serious adverse events	1 (2.2%)	0
Premature discontinuation of study drug due to an adverse event	0	0
Any adverse event	42 (91%)	26 (84%)
Treatment-related adverse event	12 (26%)	13 (42%)

Data are n (%) of patients. The data in this table reflect safety reporting from the subgroup of women patients who received treatment for up to 1 year in both the parent and extension studies

## Discussion

As previously reported, in this multicenter, randomised, blinded, placebo-controlled study in which patients with CKD and metabolic acidosis were treated for up to 52 weeks, veverimer, a novel non-absorbed HCl binder, effectively treated metabolic acidosis and improved both patient-reported limitations in ability to perform daily activities and directly measured physical performance [12]. In this pre-specified subgroup analysis of 77 women enrolled in this trial, we found that the patients in the veverimer-treated group, compared with the placebo group, had greater improvement in serum bicarbonate and physical function. These findings were consistent with the improvements observed in the overall study population. Compliance with study treatment was high and veverimer was well-tolerated in women as well as in the overall study population. No patients in the veverimer group discontinued treatment due to AEs. The overall safety profile was similar to placebo.

Although this trial was not specifically designed to evaluate mortality or progression of CKD, we noted fewer fatal events and AEs related to worsening kidney function in the veverimer group compared with the placebo group in the overall study population.

In this study, there was both an increase in serum bicarbonate and on both patient-reported and directly measured physical function. The relationship between these biochemical and clinical effects of veverimer are likely complex. Previous studies have shown that in CKD there is considerable retention of acid, some of which is reflected in alternative markers such as urinary citrate, that occurs prior to a clinically visible drop in serum bicarbonate concentration. It is plausible, therefore, that acid retention may contribute to deterioration in physical function prior to a measurable decline in serum bicarbonate. Other studies have shown that serum anion gap, or venous pH may modulate the association between serum bicarbonate levels and adverse outcomes [15, 16].

The ability to stand up from a seated position and to perform other activities of daily living are relevant patient-centric outcomes. For patients whose abilities decline, there may be important negative consequences on health and social functioning. There may also be an economic burden to patients and their families, if patients are no longer able to live independently. Metabolic acidosis causes muscle protein degradation/catabolism; and, conversely, when metabolic acidosis is treated, protein degradation declines [17, 18]. Recent studies have found an association of metabolic acidosis to frailty, fractures, and failure to thrive in patients with CKD [19, 20]. These adverse effects may be more consequential for women. In our study, we found that treatment of metabolic acidosis with veverimer improved physical function in patients with CKD in a statistically and clinically significant manner. The improvement on the KDQoL-PFD in the veverimer group (+ 11.4 points in the overall study population and + 13.2 points in the subgroup of women) was greater than 3 to 5 points, the change that has been reported as the minimal clinically-important difference for this KDQoL subscale [21–23]. Likewise, the improved physical performance in the veverimer group on the repeated chair stand test (− 4.3 s in the overall population and − 4.2 s in the subgroup of women) exceeded the 1.7 s minimal clinically-important difference for this measure [24]. These data can be considered in the context of physical function loss associated with aging. The chair stand time decrease of 4.3 s in the veverimer group between baseline and Week 52 was larger than the 3.4 s difference in mean expected performance between 80- to 89-year-olds and 60- to 69-year-olds (i.e., ~ 20-year age difference) [25]. Although we did not evaluate the mechanistic basis of physical function improvements, we found that improvements reported by patients were most frequently in the items relating to activities for which lower extremity strength is important such as walking. The repeated chair stand test is also a measure, in part, of lower extremity strength. These findings are consistent with the expected clinical manifestations of reduced muscle protein catabolism. Our findings differ from the negative findings of the BICARB study, a multicenter, randomized, placebo-controlled trial of the effect of sodium bicarbonate in patients with CKD and metabolic acidosis on physical function as assessed by the Short Physical Performance Battery (which included the repeated chair stand test) at 1-year as the primary outcome [26]. While cross-study comparisons are difficult, it is plausible that the higher rates of adverse effects reported in the sodium bicarbonate arm, particularly with respect to cardiac disorders and respiratory disorders, may have offset any beneficial effects of treatment of metabolic acidosis on physical function.

Metabolic acidosis in patient with CKD is currently managed on the principles of decreasing metabolic acid production by increasing dietary base-producing (e.g., vegetables and fruits) and/or by neutralizing acid with supplemental alkali salts such as sodium bicarbonate [27–30]. Based on evidence that chronic metabolic acidosis is harmful to multiple organs and systems including muscle, bone, kidneys, and heart, the international practice guidelines for nephrology (KDIGO) recommends treating patients with CKD when the serum bicarbonate is < 22 mmol/L [14]. The National Kidney Foundation’s Kidney Disease Outcomes Quality Initiative (KDOQI) also states that a reasonable target for serum bicarbonate is between 24 and 26 mmol/L [31]. Even with these guideline suggestions, the percentage of patients currently treated with alkali salts is low. As an example, in the well-described cohort from the Chronic Renal Insufficiency Cohort study, only 2.7% of patients were treated with alkali despite a serum bicarbonate < 22 mmol/L [32]. Sodium bicarbonate treats metabolic acidosis by entering the systemic circulation to supply bicarbonate that neutralizes retained acid. This causes a systemic sodium load that may be contraindicated in patients with CKD as these patients often have inadequate blood pressure control, edema, or heart failure [33, 34]. It is important to note that women comprise the majority of patients hospitalized for acute decompensated heart failure [35].

A limitation of this study is that it involved a subgroup analysis that, although pre-specified, should still be considered as hypothesis generating. Further, the study population lacked significant racial heterogeneity and sex was classified by self-report. Strengths of this study include its randomized multicenter design and pre-specification of the subgroup. Previous placebo-controlled trials examining the effect of metabolic acidosis on muscle function all used sodium bicarbonate as the intervention and did not find any differences in the overall population or by sex [26, 36]. This study, using a novel method to remove acid, is therefore the first to find a positive effect on measured physical function and physical function related quality of life.

## Conclusions

In conclusion, we found that veverimer, an investigational non-absorbed polymer drug, was effective in treating metabolic acidosis in women with CKD. Women treated with veverimer reported significantly improved ability to perform daily activities; their measured physical performance also improved significantly.

## Supplementary Information

**Additional file 1: Supplemental Figure 1.** Overall study design. **Supplemental Figure 2.** Veverimer Effect on Composite Endpoint at Treatment Week 52. **Supplemental Table 1.** Kidney Disease and Quality of Life – Physical Function Domain.

## Data Availability

The data that support the findings of this study are available from Tricida but restrictions apply to the availability of these data, which were used under license for the current study, and so are not publicly available. Data are however available from the authors upon reasonable request and with permission of Tricida.

## Abbreviations

AE: Adverse event
CKD: Chronic kidney disease
eGFR: Estimated glomerular filtration rate
HCl: Hydrochloric acid
KDIGO: Kidney Disease Improving Global Outcome
KDQoL-PFD: Kidney Disease and Quality of Life - Physical Function Domain
KDOQI: National Kidney Foundation’s Kidney Disease Outcomes Quality Initiative
SAE: Serious adverse event
