# Supplementary material for: Effects of veverimer on serum bicarbonate and physical function in women with chronic kidney disease and metabolic acidosis: a subgroup analysis from a randomised, controlled trial
Source: BMC Nephrol. 2022 Feb 25;23:82. doi: 10.1186/s12882-022-02690-1 (PMC8881824; doi:10.1186/s12882-022-02690-1)
Supplement: Supplementary file 1 — Additional file 1: Supplemental Figure 1. Overall study design. Supplemental Figure 2. Veverimer Effect on Composite Endpoint at Treatment Week 52. Supplemental Table 1. Kidney Disease and Quality of Life – Physical Function Domain. [file 12882_2022_2690_MOESM1_ESM.pdf]

SUPPLEMENTAL MATERIALS

Supplemental Figure 1. Overall study design

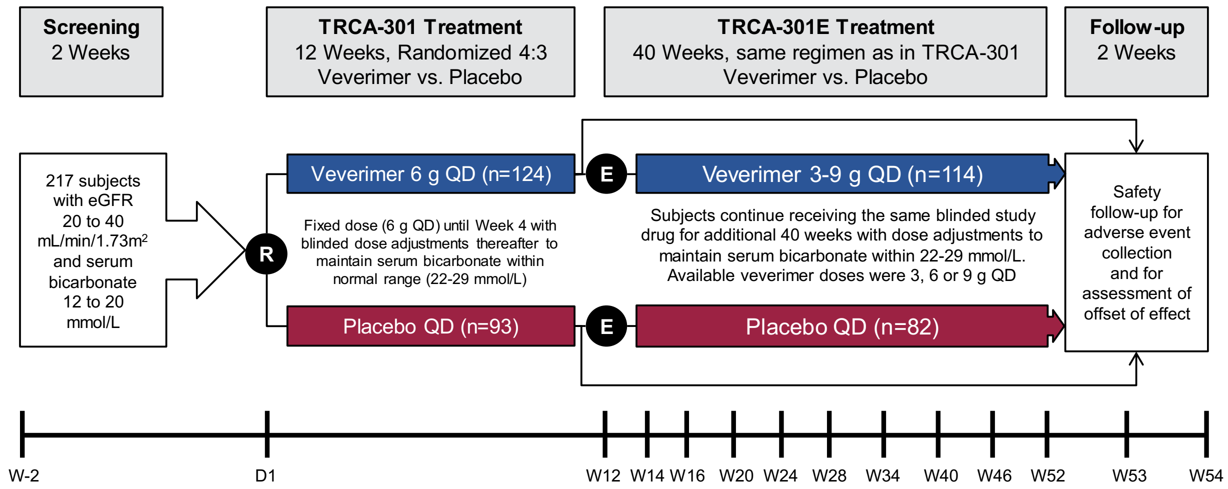

D, day; E, enrollment; eGFR, estimated glomerular filtration rate; QD, once daily; R, randomization; W, week

Supplemental Figure 2. Veverimer Effect on Composite Endpoint at Treatment Week 52

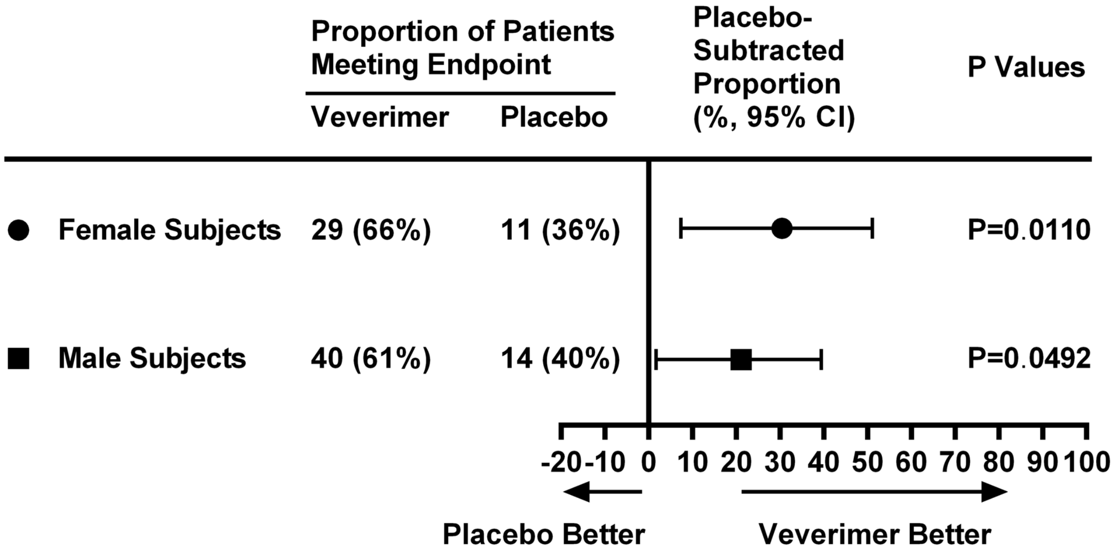

Supplemental Table 1. Kidney Disease and Quality of Life – Physical Function Domain

3. The following items are about activities you might do during a typical day.<br>Does your health now limit you in the activities? If so, how much?<br>[Mark an [illegible] in a box on each line.]		Yes, limited a lot ▼	Yes, limited a little ▼	No, not limited at all ▼
a	Vigorous activities, such as running, lifting heavy objects, participating in strenuous sports	☒	☒	☒
b	Moderate activities, such as moving a table, pushing a vacuum cleaner, bowling or playing golf.	☒	☒	☒
c	Lifting or carrying groceries	☒	☒	☒
d	Climbing several flights of stairs	☒	☒	☒
e	Climbing one flight of stairs	☒	☒	☒
f	Bending, kneeling, or stooping	☒	☒	☒
g	Walking more than a mile	☒	☒	☒
h	Walking several blocks	☒	☒	☒
i	Walking one block	☒	☒	☒
J	Bathing or dressing yourself	☒	☒	☒
